# Three New Species of *Tuber* Discovered in Alpine Fir Forests in Yunnan, China

**DOI:** 10.3390/jof10070453

**Published:** 2024-06-27

**Authors:** Lin Li, Shanping Wan, Yun Wang, Naritsada Thongklang, Mei Yang, Chengyi Liu, Zonglong Luo, Shuhong Li

**Affiliations:** 1 College of Agriculture and Biological Science, Dali University, Dali 671003, China; linlin19870311@163.com (L.L.); luozonglongfungi@163.com (Z.L.); 2 School of Science, Mae Fah Luang University, Chiang Rai 57100, Thailand; naritsada.t@gmail.com; 3 Center of Excellence in Fungal Research, Mae Fah Luang University, Chiang Rai 57100, Thailand; 4 College of Resources and Environment, Yunnan Agricultural University, Kunming 650201, China; wsp871117@163.com; 5 New Zealand Institute for Crop and Food Research Limited, Invermay Agricultural Centre, Private Bag, Mosgiel 50034, New Zealand; wangy10melrose@hotmail.com; 6 Panzhihua City Academy of Agricultural and Forestry Sciences, Panzhihua 617000, China; yangmei13550934780@163.com (M.Y.); 15729823526@163.com (C.L.); 7 Biotechnology and Germplasm Resources Institute, Yunnan Academy of Agricultural Sciences, Kunming 650223, China

**Keywords:** ITS, morphological, taxonomy, truffle, Tuberaceae

## Abstract

Three new species of *Tuber*, *T. albicavum*, *T. laojunshanense*, and *T. umbilicicavatum* belonging to the *Puberulum* phylogroup, are described based on specimens collected in alpine *Abies* forests at 3600–4000 m, Northwest Yunnan, China. *T. albicavum* is distinguished by its ascomata with a single chamber of 0.5–1.8 cm diameter, with an apical opening of 0.2–0.6 cm in diameter, and light golden-brown alveolate reticulate ascospores up to 30 μm in length; *T. laojunshanense* is characterized by having ascomata with a slightly tomentose surface, sometimes with a white navel, a relatively thick peridium, up to 280 µm, and yellow-brown spores with alveolate reticulate ornamentation, up to 34 µm in length; *T. umbilicicavatum* is characterized by smooth ascomata with a distinct white navel, a relatively thin peridium, up to 110 µm, and golden or golden-brown alveolate reticulate ascospores, up to 40 μm in length. The molecular analysis of the internal transcribed spacer region also supports that these three new species differ from previously described *Tuber* species.

## 1. Introduction

The genus *Tuber* F.H. Wigg. 1780 (Tuberaceae, Pezizales, Pezizomycotina, Ascomycota) is a significant group of fungi of economic and ecological importance. *Tuber* species are primarily distributed throughout the northern hemisphere [1,2,3,4,5], with species within the *Puberulum* phylogroup widely distributed across Europe, Asia, North America, South America, and North Africa [6]. *Tuber* species form ectomycorrhizal associations with a variety of woody plants, including those in the Fagaceae, Betulaceae, Juglandaceae, Myrtaceae, Salicaceae, Pinaceae, and Salicaceae families [7]. These associations play an important role in these forest ecosystems [8,9,10,11,12,13,14,15,16]. In recent years, new species and novel ectomycorrhizal tree partners are continually being discovered in various forest ecosystems within these regions. For example, *T. elevatireticulatum* K.F. Wong & H.T. Li, 2018, was discovered in Taiwan, China, forming ectomycorrhizal associations with *Keteleeria fortunei* var. *cyclolepis* (Flous) Silba, and a *Tuber* sp. within the *Puberulum* phylogroup has been identified in Japan as forming symbiotic relationships with *Abies sachalinensis* (F. Schmidt) Mast. [17,18]. Mature *Tuber* species have a distinctive smell that attracts small animals, aiding in spore dispersal. Additionally, some aromatic species are highly prized by humans. Notably, species such as *T. melanosporum* Vittad., 1831, and *T. magnatum* Picco, 1788, are among the most expensive delicacies in the world. Since the 1990s, species like *T. sinense* K. Tao & B. Liu, 1989, *T. sinoaestivum* J.P. Zhang & P.G. Liu, 2013, and *T. pseudohimalayense* G. Moreno, Manjón, J. Díez & García-Mont., produced in China, have become commercially significant truffles. In southwest China, the hunting and trading of these truffles have become vital sources of income. Recently, new species of *Tuber* have been discovered in southwest China, with some entering the trading market, attracting considerable attention.

Since the description of the first Chinese truffle species, *T. taiyuanense* B. Liu, in 1985, more than sixty truffle species have been reported in China [4,19,20,21], with expectations of more discoveries. This paper describes three new *Tuber* species recently found under alpine fir forests in Northwest Yunnan, China.

## 2. Materials and Methods

The specimens were collected from the alpine *Abies forrestii* var. *smithii* Viguié & Gaussen forests in Northwest Yunnan, China. These specimens were included with other studied specimens and were deposited at the BMDLU (Biological Science Museum of Dali University) and KUN-HKAS (Herbarium of Cryptogams Kunming Institute of Botany, Academia Sinica), China.

### 2.1. Morphological Study

Descriptions of microscopic and macroscopic characters were based on specimens (BMDLU L20065, L20066, L21218a, HKAS131251, 131252, 131253, 131254, 131255, 131256, 131257, 131258), following the methods of Kumar et al. [22] and Truong et al. [23], and mycorrhizal specimens (HKAS131253-ECM) following the methods of Agerer [24] and Janowski & Leski [25]. Macroscopic characters of ascomata and gleba were observed under a Nikon SMZ1000 stereo zoom microscope. The sections were made with a razorblade by hand, mounted in a 5% KOH solution or water. The sections were observed under a light microscope. The temporarily prepared microscope slides were placed under magnification up to 1000× using Nikon ECLIPSE80i (Nikon, Tokyo, Japan) compound stereomicroscope for observation and microscopic morphological photography. Measurements were made using the Image Frame work v.0.9.7. To represent variation in the size of basidiospores, 5% of measurements were excluded from each end of the range and extreme values were given in parentheses [4]. In the taxonomic descriptions of species, ‘Q (L/I)’ refers to the length/width ratio of ascospores in side-view; ‘Q_m_’ refers to the average Q of all ascospores ± standard deviation; ‘n’ refers to the number of spores measured. Key colors were obtained from Kornerup and Wanscher [26].

### 2.2. DNA Extraction, PCR Amplification, and Sequencing

Total genomic DNA was extracted from the specimen using the OMEGA Plant Genomic DNA Kit. The internal transcribed spacer (ITS) rDNA region was amplified with PCR primers ITS1F and ITS4 [23,27,28]. The large subunit nuclear ribosomal DNA (LSU) region was amplified with the PCR primers LROR and LR5 [29]. Each 30 μL PCR mixture contained 15 μL 2 × Taq Plus Master Mix II (Sangon Biotechnology Co., Kunming, China), 13 μL ddH_2_O, 0.5 μL 10 μM of forward and reverse primers, 1 μL DNA. PCR reactions were performed on a BIO-RAD C1000TM instrument. Thermal cycles with the following settings: initial denaturation for 5 min at 94 °C, followed by 32 cycles of 40 s denaturation at 94 °C, annealing at 56 °C for 40 s for ITS, and 52 °C for 30 s for LSU, extension for 1 min at 72 °C, and final extension at 72 °C for 10 min. The PCR products were verified on 1% agarose electrophoresis gels stained with ethidium bromide. The purification and sequencing of the PCR products was conducted by Sangon Biotech Limited Company (Shanghai, China).

### 2.3. Sequence Alignment and Analysis

**Figure 1:**
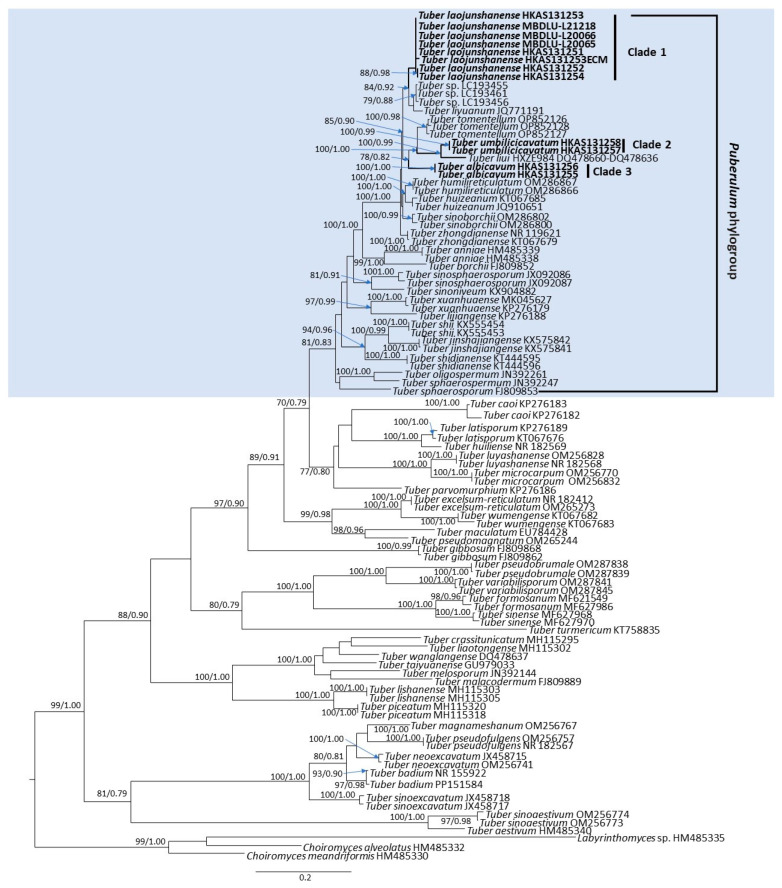
Phylogeny derived from a maximum likelihood (ML) analysis of the nrDNA-ITS sequences from *Tuber* species, using *Choiromyces alveolatus*, *C. meandriformis*, and *Labyrinthomyces* sp. as outgroup. Values next to nodes reflect maximum likelihood bootstrap support values (BS), left, and Bayesian posterior probabilities (PP), right. Names of novel species and samples with newly generated sequences are in bold.

ITS was used for the analysis of *Tuber* species diversity in this study because they appear as a useful locus for the delimitation of the genus. Ninety-nine ITS sequences from NCBI and this study representing 54 species of *Tuber* (Table 1), including *Labyrinthomyces* sp., *Choiromyces alveolatus*, and *Choiromyces meandriformis* as outgroups (Figure 1). All ITS sequences were extracted from ascomata of *Tuber* specimens except one extracted from ECM. Sequences of *Tuber* species generated in this study were submitted to the GenBank database. We first edited the sequences using BioEdit v. 7 [30], then used the basic local alignment search tool for the GenBank database to recheck whether the newly generated sequences were amplified DNA from contaminant or not and examined clusters with closely related sequences. DNA sequences were retrieved and assembled using SeqMan. Sequences were aligned using MAFFT version 7 [31]. Maximum likelihood (ML) analysis was performed using RAxML-HPC2 v. 8.2.12 [32] as implemented on the Cipres portal [33], with the GTR + G + I model and 1000 rapid bootstrap (BS) replicates for all genes. A reciprocal 70% bootstrap support approach was used to check for conflicts between the tree topologies from individual genes. As the topology of the ML tree and the Bayesian tree are similar, the ITS1, ITS2, and 5.8S sequences were combined using SequenceMatrix [34], partitioned phylogenetic analyses. For Bayesian inference (BI), the best substitution model for each partition was determined by MrModeltest 2.2 [35]. The results suggested that ITS1: JC + I, 5.8S: GTR + G + I, ITS2: K80 + I + G. Bayesian analysis was performed using MrBayes ver. 3.2.7a [36] on the Cipres [33]; four parallel runs were performed for 10 million generations sampling every 100th generation for the single gene trees. Parameter convergence > 200 was verified in Tracer v. 1.7 [37]. The phylogenetic clade was strongly supported if the bootstrap support value (BS) was ≥70% and/or a posterior probability (PP) < 0.01.

## 3. Results

### 3.1. Phylogenetic Analysis

The ML and Bayesian analyses of the 99 ITS sequences are shown in Figure 1 with associated bootstrap supports for branches.

In the phylogenetic tree, the 99 ITS sequences from *Tuber* ascomata revealed the phylogenetic relationship of 54 species: Clade 1 includes seven sequences of new species *T. laojunshanense* ascomata and one sequence of ectomycorrhizae formed by *T. laojunshanense* and *Abies forrestii* var. *smithii* from China. Clade 2 includes two sequences of new species *T. umbilicicavatum* from China. Clade 3 includes three sequences of new species *T. albicavum* from China. They belong to the *Puberulum* phylogroup. We selected the sequences of similar species of the genus *Tuber* distributed in China, and the sequences of species belonging to the *Puberulum* phylogenetic group for phylogenetic analysis with our collected specimens. The phylogenetic analysis showed that the new species are distinct from other *Tuber* species. In addition to the ITS sequences used in this phylogenetic analysis, the LSU sequences were amplified from the newly supplemented specimens in this study and uploaded to NCBI for future study.

### 3.2. Taxonomy

**Figure 2:**
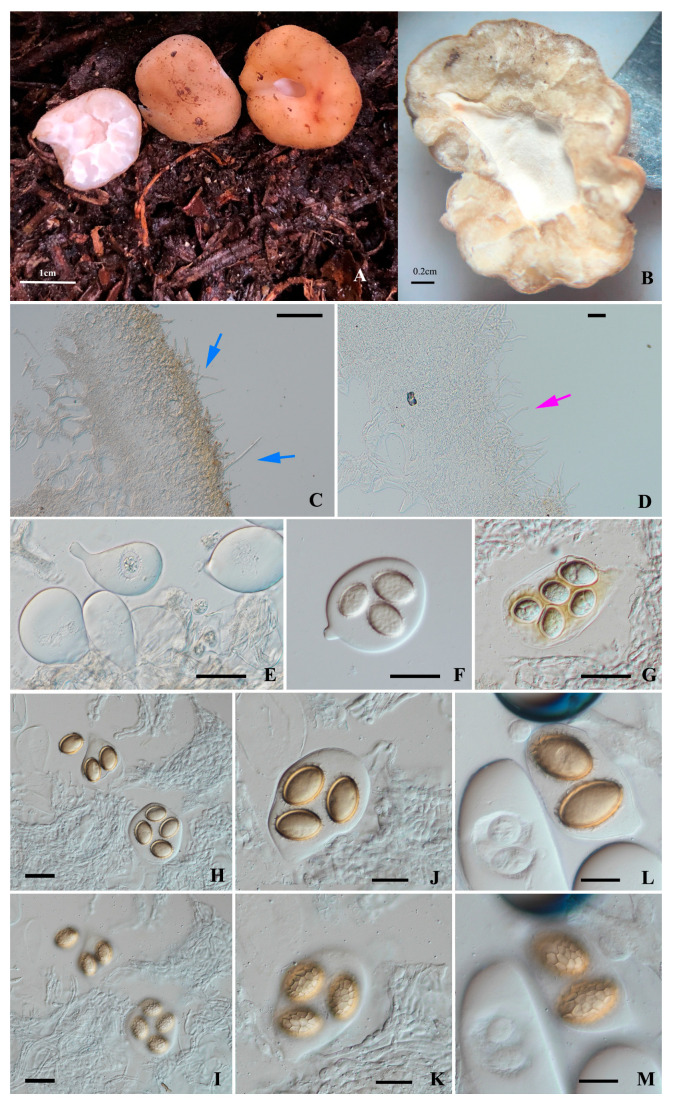
*Tuber albicavum*, (**A**) ascomata; (**B**) cross-section of dried ascomata showing gleba and cavity; (**C**) a piece of section of the peridium in 5%KOH, the blue arrows indicating bristle-like hyphae; (**D**) a cavity section in 5%KOH, pink arrow indicating hyphae extending beyond the chamber surface; (**E**) asci in 5%KOH; (**F**–**M**) ascospores and ascus. Scale bars: A = 1 cm; B = 0.2 cm; C = 50 μm; D = 10 μm; E–G = 30; H–K = 20; L–M = 30 μm.

***Tuber albicavum*** Y Wang, S.H. Li & L. Li sp. nov Figure 2.

MycoBank MB 851760

Diagnosis: Differs from other *Tuber* spp. by its almost single chamber ascomata, 0.5–1.8 cm diam., with an apical opening of 0.2–0.6 cm in diam., and light golden-brown alveolate reticulate ascospores up to 30 μm length.

Etymology: albicavum, refers to the ascomata having a white interior chamber.

Holotype: China, Yunnan, Lijiang, Jiuhe Town, 26°38′ N 99°43′ E, alt. 3753.4 m, in a forest of *Abies forrestii* var. *smithii*, 19 September 2021, Lin Li, HKAS 131256 (GenBank: ITS = PP151577 LSU = PP151587).

Ascomata subglobose, 2.0–3.5 cm diam., surface even and finely tomentose, light cinnamon to light khaki (6C7) when fresh; with a single chamber formed by the base depression, 0.5–1.8 cm diam., with an apical opening of 0.2–0.6 cm in diam., a white fluffy inner surface of the chamber; a little elastic and crisp. Gleba white (4B1) when immature, becoming khaki (6D4) at maturity, marbled with a few whitish veins. Odor: pleasant.

Peridium 80–140 µm thick, composed of two layers: outer layer pseudoparenchymatous, 27.0–62.5 µm thick, composed of subglobose to subangular cells of 6.5–14.0(–18.0) μm wide, hyaline, thin-walled; the cells in the outermost layer expand into bristle-like outer hyphae, 0.5–1.0 µm diam. at the broadest part of the base, needle-like heads, some are perpendicular to the surface, some are intertwined and prostrate, and occasionally with yellow-brown (5B4) pigment; the inner layer consists of hyaline interwoven hyphae, 29.6–74.4 µm thick, the boundary between the inner and outer layers gradually transitions, with the cells of the outer layer becoming smaller. The interior of the chamber is composed of hyaline interwoven hyphae, 50.2–80.6 µm thick, many hyphae extend beyond the surface, giving it a white, fluffy appearance, outer hyphae stubby, blunt head, with septa, occasionally forked, 45.2–76.2 µm long, 1.0–1.5 µm diam.

Asci pyriform, broadly clavate or subglobose, sometimes with short stalk, thin-walled, 45.9–61.0 × 27.5–34.6 µm, 1–4(–5)-spored.

Ascospores broadly ellipsoid, at first hyaline, becoming light golden-brown (6C8) at maturity, reticulate, thin walled 1–1.6 µm thick; dimension ranges (excluding ornamentation) 22.0–30.0 × 14.5–17.5 μm (in 1-spored asci), Q (L/I) = 1.49–2.03 Q_m_ = 1.66 ± 0.19 (n = 30), 18.5–22.5 (–24.5) × 12.0–13.5 μm (in 2-spored asci), Q (L/I) = 1.52–1.68 Q_m_ = 1.63 ± 0.06 (n = 30), (17.5–) 19.5–21.0 × 11.5–14.0 μm (in 3-spored asci), Q (L/I) = 1.50–1.67 Q_m_ = 1.60 ± 0.06 (n = 30), 18.5–20.0 (–22.5) × 11.0–13.0 μm (in 4-spored asci), Q (L/I) = 1.60– 1.69 Q_m_ = 1.65 ± 0.02 (n = 30), 15.5–18.0 (–20.0) × 9.0–11.0 μm (in 5-spored asci), Q (L/I) = 1.37–1.84 Q_m_ = 1.66 ± 0.15 (n = 10); ornamentation consists of irregular quadrilateral or pentagonal or hexagonal alveolate reticulum, the mesh 3–5 × 1–3 µm, 1–2 µm deep, 3–4 meshes across the spore width.

Ecology and distribution: Hypogeous, solitary, or in groups in the soils under the forest of *Abies forrestii* var. *smithii*, alt. 3700–3800 m, fruiting from autumn. Known only from Yunnan Province, China.

Additional specimen examined: China, Yunnan Province, Lijing, Jiuhe Town, 26°29′ N, 99°39′ E, alt. 3846 m, 12 September 2020, Lin Li (GenBank: HKAS131255 ITS = PP151578 LSU = PP151588).

Edibility: fragrant, edible.

Notes: The phylogenetic tree shows that *Tuber albicavum* is closely related to the known species *T. tomentellum*, *T.liui*, and a new species reported in this study, *T. umbilicicavatum*, forming the same clade. Compared to them, firstly, in terms of macroscopic characteristics, *T. albicavum* has a basal depression that forms a cavity, while *T. umbilicicavatum* only presents a navel-like depression. The ascomata of *T. tomentellum* are merely described as having ‘an indistinctly basal depression’ [21,58], and ascomata of *T. liui* have grooves and very small pores, with white soft hairs within the grooves [58]. Secondly, the ascomata surface of *T. albicavum* is even and finely tomentose, which is similar to *T. tomentellum*, but *T.liui* and *T. umbilicicavatum* have smooth ascocarp surfaces. Additionally, both *T. tomentellum* and *T. albicavum* are found in Yunnan Province, China, but *T. tomentellum* is distributed in *Pinus* forests at altitudes not exceeding 2000 m [21], while *T. albicavum* is found in *Abies forrestii* var. *smithii* forests at altitudes of 3800–3900 m. *T. liui*, which also occurs in high-altitude regions (3100 m), is found in the alpine *Quercus aquifolioides* Rehder & E. H. Wilson forests [58]. Molecular analysis also shows that *T. albicavum* is separated from other *Tuber* species, they were divided into different species with a high support rate.

**Figure 3:**
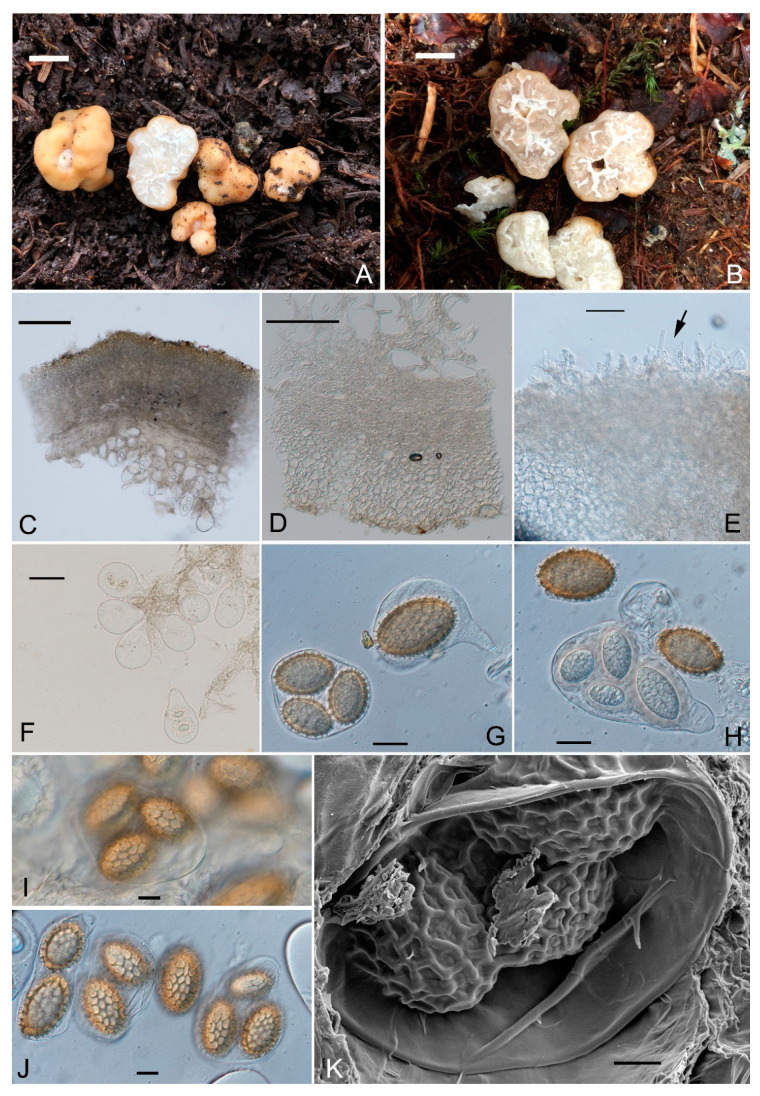
*Tuber laojunshanense*, (**A**,**B**) ascomata and gleba; (**C**,**D**) a piece of the section of peridium in 5% KOH; (**E**) peridium outer layer in 5% KOH, the black arrows indicating hyphae extending beyond the surface; (**F**) asci with immature ascospores in 5%KOH; (**G**) ascus contains 1 spore or 3 spores when mature; (**H**) released ascospores and asci; (**I**,**J**) asci and ascospores; (**K**) SEM ascospores (dry sample). Scale bars: A,B = 1 cm; C,D = 100 μm; E = 10 μm; F = 50 μm; G–K = 10 μm.

***Tuber laojunshanense*** Y Wang, S.H. Li & L. Li sp. nov Figure 3.

Mycobank number: MB 851752

Diagnosis: Differs from other *Tuber* spp. by its ascomata which has a slightly tomentose surface, sometimes with a white navel, a relatively thick peridium up to 280 µm, and yellow-brown spores with alveolate reticulum patterns, up to 34 µm in length.

Etymology: laojunshanense, refers to the type locality of the Mt. laojunshan.

Holotype: China, Yunnan, Lijiang, Juhe Town, 26°37′ N 99°43′ E, alt. 3856 m, in the forest of *Abies forrestii* var. *smithii*. 16 September 2022, Lin Li, HKAS 131253 (GenBank: ITS = PP151583 LSU = PP151593).

Ascomata subglobose or irregular in form, occasionally irregularly lobed with furrows on the surface, slightly tomentose, 1.0–3.5 cm diam, sometimes with a white navel, light khaki (4C6) when fresh. Gleba white (4B1) when immature, becoming brown (4D4) at maturity, marbled with whitish veins. Odor light scent.

Peridium 160–280 µm thick, composed of two layers: outer layer pseudoparenchymatous, 50–110 µm thick, composed of subglobose to subangular cells of 3.5–18.0(–20.0) μm wide, hyaline, thin-walled, the cells in the outermost layer expanding into bristle-like outer hyphae, 0.5–1.0 µm diam., irregularly arranged, either interwoven or prostrate, stubby, blunt head, with septa, occasionally with yellow-brown (4E8) pigment; inner layer consists of hyaline interwoven hyphae, 80–160 µm thick, the boundary between the inner and outer layers is gradually transitioned by the cells of the outer layer becoming smaller.

Asci pyriform, broadly clavate or subglobose, sometimes with short stalk, thin-walled, 30.5–50.0 × 29.5–39.5 µm, 1–3(-4)-spored.

Ascospores ellipsoid or broadly ellipsoid, at first hyaline, becoming yellow brown (5C8) at maturity, reticulate, thin walled 1–2 µm thick; dimension ranges (excluding ornamentation) are 28.0–34.0 × 19.5–21.0 μm (in 1-spored asci), Q (L/I) = 1.34–1.85 Q_m_ = 1.64 ± 0.05 (n = 30), 26.0–29.5 × 17.0–20.5 μm (in 2-spored asci), Q (L/I) = 1.25–1.88 Q_m_ = 1.59 ± 0.03 (n = 30), (19.5–) 24.0–28.5 × (10.5–) 15.0–18.0 μm (in 3-spored asci), Q (L/I) = 1.27–1.84 Q_m_ = 1.56 ± 0.04 (n = 30), 11.5–16.5 × 11.5–8.0 μm (in 4-spored asci), Q (L/I) = 1.30–1.76 Q_m_ = 1.48 ± 0.09 (n = 11); ornamentation consists of regular pentagonal or hexagonal alveolate reticulum, reticulum 1–3 μm high, mostly 3–5 meshes across the spore width.

**Figure 4:**
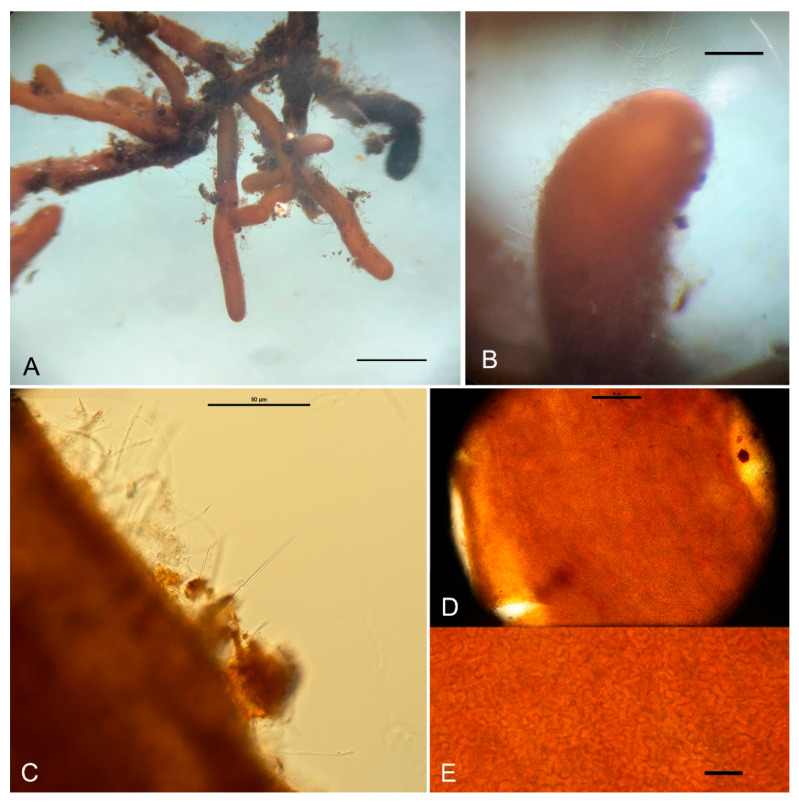
Ectomycorrhizae of *T. laojunshanense* with *Abies forrestii* var. *smithii*. (**A**) Mycorrhizal clusters; (**B**) a mycorrhizal tip with spiky cystidia; (**C**) spiky cystidia arising from the cells of the outer mantle layer; (**D**,**E**) mantle surface structure. Scale bars: A = 0.5 cm; B = 1 mm; C,D = 50 μm; E = 20 μm.

Ecology and distribution: Hypogeous, solitary, or groups in the soil under the forest of *Abies forrestii* var. *smithii*, alt. 3600–3900 m, fruiting in autumn. It forms ectomycorrhizae (ECM) with *A. forrestii* var. *smithii* (Figure 4). ECMs have simple ramified systems, in a monopodial-pinnate pattern; up to 8.0 mm long, 2.0 mm wide, light yellow–ocher (5C8-7E7), unramified ends up to 3.0 mm long, 0.4–1.0 mm in diam. Mantle 10–35 μm thick, three to five layers with interlocked pseudoparenchymatous surface. Cystidia needle-like, smooth, colorless, nonseptated or monoseptated. Hartig nets palmetii and single hyphal rows.

Found only in Yunnan Province, southwestern China.

Additional specimen examined: China, Yunnan Province, Lijing, Jiuhe Town, 26°27′ N 99°37′ E, alt. 3645 m, 11. Aug. 2020, Lin Li (BMDLU L20065 GenBank: ITS = PP151573, L20066 GenBank: ITS = PP151574). China, Yunnan Province, Lijing, Jiuhe Town, 26°29′ N 99°27′ E, alt. 3875 m, 19 September 2021, Lin Li (GenBank: HKAS131251 ITS = PP151579 LSU = PP151589, HKAS131252 ITS = PP151580 LSU = PP151590, HKAS131254 ITS = PP151581 LSU = PP151591, BMDLU-L21218a ITS = PP151582 LSU = PP151592). China, Yunnan Province, Lijing, Jiuhe Town, 26°37′ N 99°43′ E, alt. 3856 m, at the root tips of *Abies forrestii* var. *smithii*, 16 September 2022, Lin Li (BMDLU L22070 ECM isolate GenBank: ITS = PP124613).

Edibility: fragrant, edible.

Notes: The phylogenetic tree shows that *Tuber. laojunshanense* is closely related to *T. liyuanum* and a potential *Tuber* species found in Taiwan, forming the same clade. When comparing the two, *T. liyuanum* [47] and *T. laojunshanense* have similar colored ascomata, which are light brown or light khaki, with a similar surface characterized by shallow irregular fissures and slight tomentose cover. They also have a similar peridium thickness and a two-layered structure. However, the obvious differences are that *T. liyuanum* has larger ascospores reaching 60 µm in length, while *T. laojunshanense* has smaller ascospores, only up to 34 µm in length. Furthermore, *T. liyuanum* has a strong or pungent but pleasant scent when fresh, whereas *T. laojunshanense* has a light pleasant scent when fresh. Additionally, both *T. liyuanum* and *T. laojunshanense* are found in Yunnan Province, China, but *T. liyuanum* is distributed in *Pinus yunnanensis* Franch. forests at altitudes not exceeding 2000 m [47], while *T. laojunshanense* is found in *Abies forrestii* var. *smithii* forests at altitudes of 3600–3800 m. Molecular analysis also shows that *T. laojunshanense* is separated from other *Tuber* species; they were divided into different species with a high support rate.

**Figure 5:**
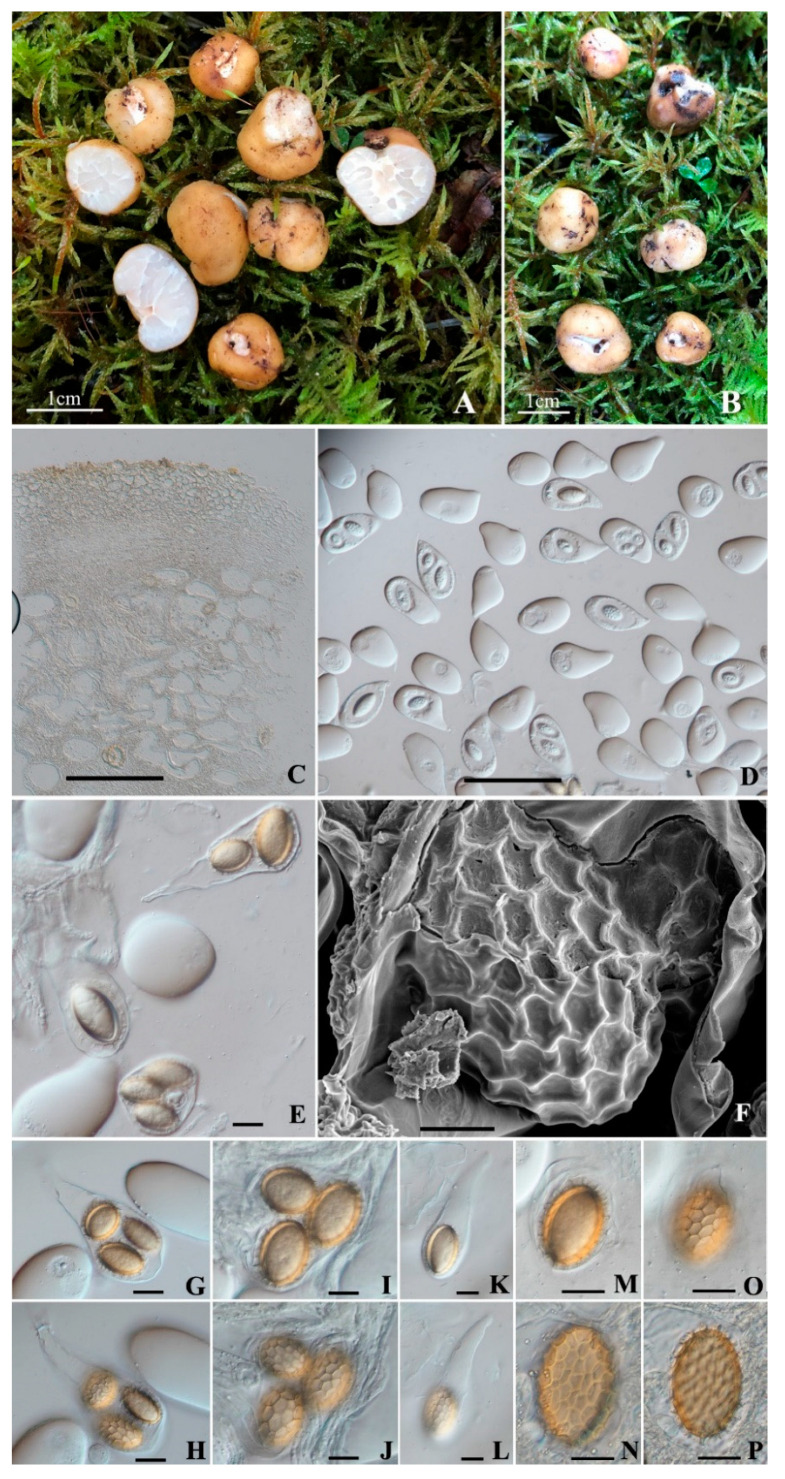
*Tuber umbilicicavatum*, (**A**,**B**) ascomata; (**C**) a piece of the section of the ascomata in 5%KOH; (**D**) asci in lactophenol; (**E**) 1-spored, 2-spored, and 4-spored asci; (**F**) SEM ascospores (dry sample); (**G**–**P**) ascospores and ascus; Scale bars: A,B = 1 cm; C,D = 100 μm; E,G–P = 20 μm; F = 10 μm.

***Tuber umbilicicavatum*** Y Wang, S.H. Li & L. Li sp. nov Figure 5.

MycoBank MB 851759

Diagnosis: Differs from other *Tuber* spp. by its smooth ascomata with a distinct white navel, a relatively thin peridium up to 110 µm, and golden or golden-brown alveolate reticulate ascospores up to 40 μm length.

Etymology: umbilicicavatum, refers to the ascomata having a white navel.

Holotype: China, Yunnan, Lijing, Jiuhe Town, 26°37′ N 99°43′ E, alt. 3870.7 m, in a forest of *Abies forrestii* var. *smithii*, 19 September 2021, Lin Li, HKAS 131258 (GenBank: ITS = PP151575 LSU = PP151585).

Ascomata subglobose or irregular in form, 0.5–1.5 cm diam., with a distinct white navel, light cinnamon (5D8) when fresh, poor elasticity, brittle, easy to crack when pressed by hand, smooth on the surface. Gleba white (4B1) when immature, lighter brown (4D6) at maturity, marbled with a few whitish veins. Odor is pleasant.

Peridium 80–110 µm thick, composed of two layers: outer layer pseudoparenchymatous, 29.5–47.5 µm thick, composed of subglobose to subangular cells of 8.5–30 μm wide, hyaline, thin-walled, occasionally cells of the outermost layer with light brown (4B6) pigment; inner layer of hyaline interwoven hyphae, 27.7–52.2 µm thick, the boundary between the inner and outer layers is gradually transitioned by the cells of the outer layer becoming smaller.

Asci pyriform, broadly clavate or subglobose, sometimes with short stalk, tubular links were not observed, thin-walled, 52.0–67.5 × 31.6–40.4 µm, 1–3(–4)-spored.

Ascospores ellipsoid or broadly ellipsoid, at first hyaline, becoming golden or golden-brown (5B8) at maturity, reticulate, thin walled 0.9–1.7 µm thick; excluding ornamentations, dimension ranges (excluding ornamentation) are (34.0–) 38.5–40.5 × 20.0–26.5 μm (in 1-spored asci), Q (L/I) = 1.54–1.67 Q_m_ = 1.63 ± 0.05 (n = 30), 35.0–38.5 (–40.0) × 19.0–22.5 μm (in 2-spored asci), Q (L/I) = 1.56–1.74 Q_m_ = 1.68 ± 0.06 (n = 30), (27.5–) 31.0–35.5 × 19.0–24.0 μm (in 3-spored asci), Q (L/I) = 1.57–1.66 Q_m_ = 1.59 ± 0.18 (n = 30), 28.5–32.5 × 18.0–20.5 μm (in 4-spored asci), Q (L/I) = 1.54–1.69 Q_m_ = 1.67 ± 0.14 (n = 11); ornamentation consists of regular quadrilateral or pentagonal alveolate reticulum, the mesh 2.5–6 × 1.5–3.5 µm, 2–3 µm deep, 3–5 meshes across the spore width.

Ecology and distribution: Hypogeous, solitary, or in groups in the soils under the forest of *Abies forrestii* var. *smithii*, alt. 3800–3900 m fruiting in autumn. Known only from Yunnan Province, China.

Additional specimen examined: China, Yunnan Province, Lijing, Jiuhe Town, 26°29′ N, 99°39′ E, alt. 3916 m, 19 September 2021, Lin Li (GenBank: HKAS131257 ITS = PP151576 LSU = PP151586).

Edibility: fragrant, edible.

Notes: The phylogenetic tree shows that *Tuber umbilicicavatum* is closely related to *T. liui* and *T. tomentellum*, forming the same clade. Comparing the three, all have small ascomata not exceeding 3 cm, but the distinguishing feature of *T. umbilicicavatum* is its smooth surface with a distinct navel-like depression. In contrast, *T. liui* also has a smooth surface but with grooves and very small pores, and the grooves contain white soft hairs [58], *T. tomentellum* has a tomentose surface with an indistinct navel [21]. Additionally, the ascospores of *T. umbilicicavatum* are smaller, up to 40 μm in length, whereas *T. tomentellum* ascospores can be 70 μm in length [16], and *T. liui* ascospores can reach 78 (–94) μm in length [58]. Furthermore, *T. tomentellum* is distributed in *Pinus* forests at an altitude of around 2000 m in central Yunnan [21], *T. liui* is found in *Quercus aquifolioides* forests at an altitude of 3100 m, and *T. umbilicicavatum* is distributed in *Abies forrestii* var. *smithii* forests at altitudes of 3800–3900 m. Molecular analysis also shows that *T. umbilicicavatum* is separated from other *Tuber* species; they were divided into different species with a high support rate.

## 4. Discussion

Since the first *Tuber* species was documented in China in 1985, more than sixty species have been reported, with half of these being newly identified by science [4,20,21]. The great majority of these species are found in southwest China (Yunnan, Sichuan, and Xizang Province) implying southwest China might be one of the epicenters for the evolution of *Tuber* species. Quite a few alpine *Tuber* species have been found in southwest China, which further supports this speculation. *T. liui* was the first alpine species found in the *Quercus aquifolioides* forest at alt. 3100 m, Xizang of China [58]. *T. zhongdianense* was the second one discovered at 3400 m in *Quercus monimotricha* bush in Yunnan, China [59]. *T. albicavum*, *T. laojunshanense*, and *T. umbilicicavatum* were recently found under the alpine fir forests in northwest Yunnan, growing with *Abies forrestii* var. *smithii* at even higher altitudes between 3600 and 4000 m. All five species belong to the *Puberulum* phylogroup sharing the same morphological features: smaller light-colored ascomata, double-layer peridium, and alveolate reticulum ascospores. Phylogenetic analysis showed all the alpine species were grouped, indicating they are closely related in phylogeny. The unique climate of the alpine zone forests in southwest China nurtured these truffles and made them differ from other *Tuber* species.

In a molecular-based study on the symbiotic tree partners of *Tuber* species [7], 16 European *Tuber* species were analyzed, including 156 ECM symbionts formed by *Tuber* species. None of the *Tuber* species were found to be exclusively associated with trees of the Pinaceae family, reflecting the diversity of the mycorrhizal tree partners among the *Tuber* species. The study also revealed that, for species within the *Puberulum* phylogroup (specifically *T. borchii* and *T. anniae*), approximately 30% of ECM symbionts were formed with coniferous trees (Pinaceae), while about 70% were formed with broad-leaved trees [7].

The three new species reported in this paper also belong to the *Puberulum* phylogroup, and the habitats of the studied specimens are similar. Although these species were confirmed to be associated with *Abies forrestii* var. *smithii*, it is known that the same *Tuber* species can form symbiotic mycorrhizae with different trees in various habitats, including both coniferous and broad-leaved trees. Therefore, it cannot be conclusively stated that these species form mycorrhizal associations exclusively with *Abies forrestii* var. *smithii*. Nevertheless, these truffles play an important role in the alpine forest ecosystems, symbiotically associated with their trees, and provide food to animals dwelling in these forests.

## 5. Conclusions

Based on morphological and DNA sequence evidence, this study describes three new species of white truffles, *T. albicavum*, *T. laojunshanense*, and *T. umbilicicavatum*, collected from alpine fir forests in Yunnan, China, as belonging to the *Puberulum* phylogroup. These species represent new scientific records of *Tuber* species distributed at elevations of 3600–4000 m.

## Figures and Tables

**Table 1:** Taxa information and GenBank accession numbers of the sequences used in this study. The newly generated sequences are in bold.

Species Name	Voucher	Origin	GenBank No.	References
*Choiromyces alveolatus*	MES97	USA	HM485332	[38]
*Choiromyces meandriformis*	RH691	USA	HM485330	[38]
*Labyrinthomyces sp*	JT27750	Australia	HM485335	[38]
*Tuber aestivum*	JT30500	Japan	HM485340	[38]
*Tuber albicavum*	HKAS131256 *	China	PP151577	This study
*Tuber albicavum*	HKAS131255	China	PP151578	This study
*Tuber anniae*	JT13209	Japan	HM485338	[38]
*Tuber anniae*	JT22695	Japan	HM485339	[38]
*Tuber badium*	HKAS 88789	China	NR_155922	[39]
*Tuber badium*	BMDLU-L3152	China	PP151584	This study
*Tuber borchii*	GB1/GB32	Italy	FJ809852	[40]
*Tuber caoi*	BJTC FAN271	China	KP276183	[41]
*Tuber caoi*	BJTC FAN293	China	KP276182	[41]
*Tuber crassitunicatum*	BJTC FAN465	China	MH115295	[42]
*Tuber excelsum-reticulatum*	BJTC FAN863	China	NR_182412	[4]
*Tuber excelsum-reticulatum*	BJTC FAN864	China	OM265273	[4]
*Tuber formosanum*	BJTC FAN107	China	MF621549	[43]
*Tuber formosanum*	BJTC FAN356	China	MF627986	[43]
*Tuber gibbosum*	JT30580	USA	FJ809868	[40]
*Tuber gibbosum*	JT26632	USA	FJ809862	[40]
*Tuber huiliense*	BJTC FAN288	China	NR_182569	[4]
*Tuber huizeanum*	BJTC FAN314	China	KT067685	[44]
*Tuber huizeanum*	BJTC FAN186	China	JQ910651	[44]
*Tuber humilireticulatum*	BJTC FAN189	China	OM286867	[4]
*Tuber humilireticulatum*	BJTC FAN174	China	OM286866	[4]
*Tuber jinshajiangense*	BJTC FAN406	China	KX575841	[45]
*Tuber jinshajiangense*	BJTC FAN407	China	KX575842	[45]
*Tuber laojunshanense*	HKAS131253 *	China	PP151583	This study
*Tuber laojunshanense*	BMDLU-L20065	China	PP151573	This study
*Tuber laojunshanense*	BMDLU-L20066	China	PP151574	This study
*Tuber laojunshanense*	HKAS131251	China	PP151579	This study
*Tuber laojunshanense*	BMDLU-L22070ECM	China	PP124613	This study
*Tuber laojunshanense*	HKAS131252	China	PP151580	This study
*Tuber laojunshanense*	HKAS131254	China	PP151581	This study
*Tuber laojunshanense*	BMDLU-L21218a	China	PP151582	This study
*Tuber latisporum*	BJTC FAN125	China	KT067676	[44]
*Tuber latisporum*	BJTC FAN126	China	KP276189	[44]
*Tuber liaotongense*	BJTC FAN550	China	MH115302	[42]
*Tuber lijiangense*	BJTC FAN307	China	KP276188	[41]
*Tuber lishanense*	BJTC FAN683	China	MH115305	[42]
*Tuber lishanense*	BJTC FAN718	China	MH115303	[42]
*Tuber liui*	HXZE 984	China	DQ478660	[46]
*Tuber liui*	HXZE 984	China	DQ478636	[46]
*Tuber liyuanum*	BJTC FAN162	China	JQ771191	[47]
*Tuber luyashanense*	BJTC FAN846	China	OM256828	[4]
*Tuber luyashanense*	BJTC FAN1031	China	NR_182568	[4]
*Tuber maculatum*	RBG Kew K(M)17936	UK	EU784428	[48]
*Tuber magnameshanum*	BJTC FAN537	China	OM256767	[4]
*Tuber malacodermum*	JT32319	Spain	FJ809889	[40]
*Tuber melosporum*	AH31737	Spain	JN392144	[49]
*Tuber microcarpum*	BJTC FAN880	China	OM256832	[4]
*Tuber microcarpum*	BJTC FAN866	China	OM256770	[4]
*Tuber neoexcavatum*	BJTC FAN316	China	OM256741	[4]
*Tuber neoexcavatum*	BJTC FAN184	China	JX458715	[4]
*Tuber oligospermum*	AH38984	USA	JN392261	[50]
*Tuber parvomurphium*	BJTC FAN298	China	KP276186	[41]
*Tuber piceatum*	HMAS 97125	China	MH115318	[42]
*Tuber piceatum*	HMAS 97124	China	MH115320	[42]
*Tuber pseudobrumale*	BJTC FAN322	China	OM287839	[4]
*Tuber pseudobrumale*	BJTC FAN306	China	OM287838	[4]
*Tuber pseudofulgens*	BJTC FAN399	China	NR_182567	[4]
*Tuber pseudofulgens*	BJTC FAN399	China	OM256757	[4]
*Tuber pseudomaganatum*	BJTC FAN391	China	OM265244	[4]
*Tuber shidianense*	HKAS 88770	China	KT444595	[51]
*Tuber shidianense*	HKAS 88771	China	KT444596	[51]
*Tuber shii*	BJTC FAN405	China	KX555453	[45]
*Tuber shii*	BJTC FAN409	China	KX555454	[45]
*Tuber sinense*	BJTC FAN108	China	MF627968	[43]
*Tuber sinense*	BJTC FAN110	China	MF627970	[43]
*Tuber sinoaestivum*	BJTC FAN522	China	OM256774	[4]
*Tuber sinoaestivum*	BJTC FAN487	China	OM256773	[4]
*Tuber sinoborchii*	BJTC FAN169	China	OM286800	[4]
*Tuber sinoborchii*	BJTC FAN171	China	OM286802	[4]
*Tuber sinoexcavatum*	BJTC FAN166	China	JX458718	[52]
*Tuber sinoexcavatum*	BJTC FAN130	China	JX458717	[52]
*Tuber sinoniveum*	HKAS 88792	China	KX904882	[53]
*Tuber sinosphaerosporum*	BJTC FAN136	China	JX092087	[54]
*Tuber sinosphaerosporum*	BJTC FAN135	China	JX092086	[54]
*Tuber sp*	GGPI1	China	LC193461	GenBank
*Tuber sp*	GGPC2	China	LC193455	GenBank
*Tuber sp*	GGPC3A	China	LC193456	GenBank
*Tuber sphaerospermum*	AH39184	USA	JN392247	[50]
*Tuber sphaerosporum*	JT12487	USA	FJ809853	[40]
*Tuber taiyuanense*	T42_HM75888	China	GU979033	[55]
*Tuber tomentellum*	BJTC FAN1330	China	OP852126	[21]
*Tuber tomentellum*	BJTC FAN1340	China	OP852127	[21]
*Tuber tomentellum*	BJTC FAN1346	China	OP852128	[21]
*Tuber turmericum*	BJTC FAN471	China	KT758835	[56]
*Tuber umbilicicavatum*	HKAS131257	China	PP151576	This study
*Tuber umbilicicavatum*	HKAS131258 *	China	PP151575	This study
*Tuber variabilisporum*	BJTC FAN330	China	OM287841	[4]
*Tuber variabilisporum*	BJTC FAN362	China	OM287845	[4]
*Tuber wanglangense*	HMAS60220	China	DQ478637	[46]
*Tuber wumengense*	BJTC FAN218A	China	KT067682	[44]
*Tuber wumengense*	BJTC FAN292	China	KT067683	[44]
*Tuber xuanhuaense*	HMAS 60213	China	KP276179	[44]
*Tuber xuanhuaense*	BJTC FAN618	China	MK045627	[44]
*Tuber zhongdianense*	HKAS:Wang-0299	China	NR_119621	[57]
*Tuber zhongdianense*	BJTC FAN178	China	KT067679	[44]

* Holotype.

## Data Availability

Data are contained within the article.
